# Draft genome sequence of biosurfactant-producing strains *Rhodococcus* sp. IITD102, *Lysinibacillus* sp. IITD104 and *Paenibacillus* sp. IITD108, isolated from oil-contaminated soil

**DOI:** 10.1128/mra.01329-24

**Published:** 2025-06-09

**Authors:** Nidhi Patil, Preeti Srivastava

**Affiliations:** 1 Department of Biochemical engineering and Biotechnology, Indian Institute of Technology Delhi 28817, New Delhi, India; California State University, San Marcos, California, USA

**Keywords:** biosurfactants, asphaltene

## Abstract

We report the draft genome sequence of three biosurfactant-producing strains *Rhodococcus* sp. IITD102, *Lysinibacillus* sp. IITD104 and *Paenibacillus* sp. IITD108. They produce a glycolipid type of biosurfactant useful in enhanced oil recovery. The nucleotide sequence will provide insights into the various genes and regulators involved in the biosynthesis of biosurfactant.

## ANNOUNCEMENT

**Fig 1:**
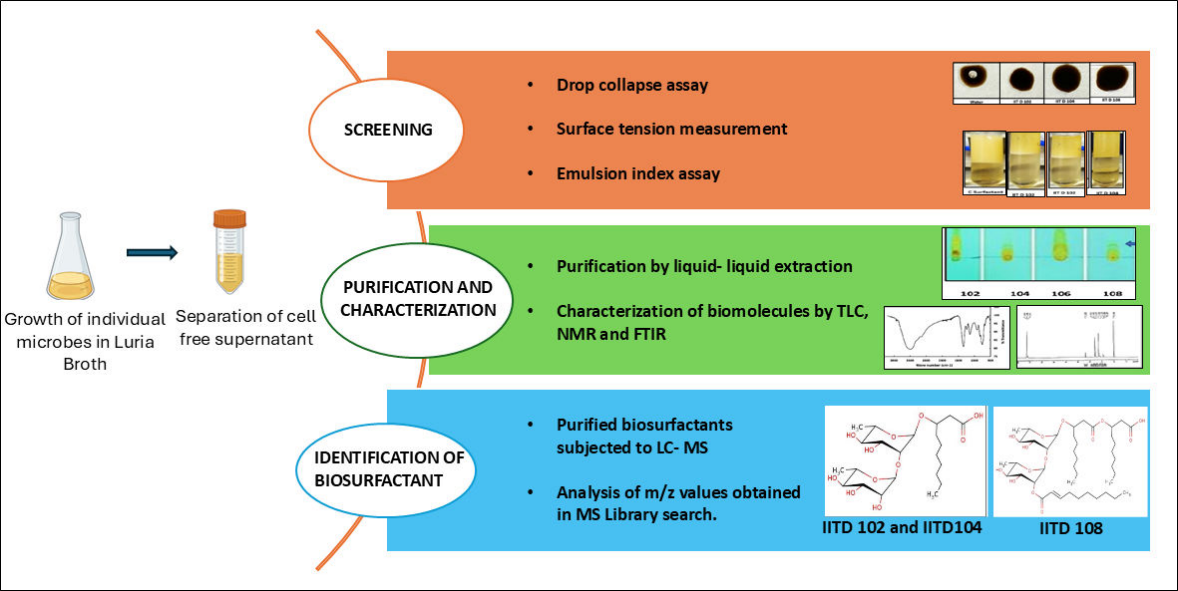
Schematic representation of the methodology used in screening biosurfactants and their characterization from the three strains.

*Rhodococcus* sp. IITD102, *Lysinibacillus* sp. IITD104, and *Paenibacillus* sp. IITD108 were isolated from crude-oil contaminated soil by enrichment culture using asphaltene as a carbon source (1). Sampling of soil was carried out in July 2013 at a depth of 10 cm in Faridabad, India at 28.40 N, 77.31 E whose elevation was 198 m. Micro-organisms were isolated by inoculating 1 g/L soil into a minimal salt medium (MSM) containing 2.5 g/L of asphaltene. The culture flasks were incubated at 30°C and 180 rpm for 10 days, and bacteria were isolated on MSM agarose (2% [wt/vol]) plates overlaid with asphaltene dissolved in toluene (75 g/L). The composition of MSM (100 mL) was 0.2 g Na_2_HPO_4_, 0.1 g KH_2_PO_4_, 0.425 g ammonium oxalate, and 0.040 g MgCl_2_ along with trace elements (KI, LiCl, MnCl_2_, H_3_BO_3_, ZnCl_2_, CoCl_2_, NiCl_2_, BaCl_2_, [NH_4_]_6_MoO_24_, SnCl_2_, and Al [OH]_3_). Colonies were obtained after 5 days of incubation and typed morphologically. Repeated rounds of isolation were carried out to obtain single pure colonies (1). *Rhodococcus* sp. IITD102 and *Lysinibacillus* sp. IITD 104 were found to produce a biosurfactant di-rhamnopyranosic hydroxydecanoic acid, while *Paenibacillus* sp. IITD108 produced 3-O-(2-O-[2E-decanoyl]-αL rhamnopyranosyl)-3-hydroxydecanoic acid (2). A schematic representation of the recommended methodology used for screening and characterization of biosurfactants in the previous study is depicted in Fig. 1 (3). The rationale for genome sequencing was to identify the genes for biosurfactant biosynthesis and regulation.

The DNeasy PowerSoil Pro kit (catalog number 47016, Qiagen) was used to isolate genomic DNA from bacteria grown on Luria broth (30°C, 180 rpm). A library was prepared using the NEBNext Ultra II FS DNA library preparation kit (catalog number E6177).

The paired-end sequencing was carried out using the Illumina HiSeq 4000 platform. Trimming and removal of adapter sequences were done with default settings using Trimgalore software, v0.6.4 (4). DeNovo assembly was carried out for IITD108, and the raw reads obtained were assembled using Unicycler version v0.4.8 with default settings (5). For reference-based assembly of trimmed reads of *Rhodococcus* sp. IITD102, Contiguator v2 was used, and for raw reads of *Lysinibacillus* sp. IITD104, Medusa v1.6 was used (6, 7). The genome assembly statistics of all three bacteria are shown in Table 1. The EZ BioCloud ANI Calculator web tool having OrthoANIu with USEARCH v8.1.1861_i86linux32 was used to identify the bacterium while comparing the two prokaryotic genomes (8). The GC content was analyzed using FastQC v0.11.2 (9). NCBI Prokaryotic Genome Annotation Pipeline (v6.6 for strain IITD102 and IITD108, v6.9 for IITD104) was used to annotate putative genes (10, 11). Genes related to biosurfactant synthesis, such as rhamnosyltransferase and glucosyltransferases, were identified in IITD102 and IITD108, respectively. Several genes encoding for acyltransferases have been found in all three strains. Genes encoding enzymes involved in aromatic hydrocarbon degradation, such as catechol 2,3-dioxygenase, alkane monooxygenase, benzene 1,2-dioxygenase, and protocatechuate 3,4-dioxygenase, among others, were also identified in these three microorganisms.

**TABLE 1:** Genome assembly statistics for the sequenced strains

Species	*Rhodococcus* sp.	*Lysinibacillus* sp.	*Paenibacillus* sp.
Strain	IITD102	IITD104	IITD108
Biosample no	SAMN39849371	SAMN39604294	SAMN39603554
SRA accession no	SRR30566949	SRR30570967	SRR30571914
Bioproject no	PRJNA1074079	PRJNA1068812	PRJNA1068804
Genbank accession no	JAZKJU000000000	JBAHYH000000000	JAZHCT000000000
Assembly size	5,955,165	4,508,837	6,743,892
Coverage	1408.4×	218.2×	200.1×
Total number of reads	27,602,842	3,226,604	4,348,005
Read length	128 (trimmed reads)	150 (raw reads)	150 (raw reads)
*N*_50_	219.9 kb	191.6 kb	164.6 kb
No of contigs	114	48	103
GC (%)	70.5	37.5	44
Total coding genes	5,394	4,422	5,605
Pseudogenes	98	61	57
rRNA	3	4	5
Percentage identity with the closest strain (OrthoANI)	99.19 (*Rhodococcus ruber* strain C1 chromosome (NZ_CP044211.1)	98.58 *Lysinibacillus boronitolerans* JCM 21713 (NZ_AVCW01000001)	67.64 *Paenibacillus* sp. UBA10396
Assembly method used	Reference-based assembly using Contiguator 2 (*Rhodococcus ruber* strain C1 chromosome (NZ_CP044211.1)	Reference-based assembly using Medusa v1.6 (CP150202.1 *Lysinibacillus* sp. FSL P2-0066 chromosome)	*De Novo* Assembly using Unicycler version v0.4.8

## Data Availability

All data associated with these genomes are deposited under NCBI BioProject numbers PRJNA1074079, PRJNA1068812, and PRJNA1068804 for IITD102, IITD104, and IITD108, respectively. See Table 1 for strain specific accessions.
